# Development and validation of a personal responsibility scale for Chinese college students

**DOI:** 10.3389/fpsyg.2023.1231462

**Published:** 2023-10-16

**Authors:** Yunzhe Ren, Jixia Wu, Hong Qin

**Affiliations:** School of Education, Soochow University, Suzhou, China

**Keywords:** responsibility, responsibility scale, social responsibility, trust, prosocial, conscientiousness

## Abstract

**Introduction:**

The complexity of the concept of responsibility has led to a relative lack of measuring tools. Meanwhile, the widely used measurement of conscientiousness in the Big Five personality traits suffers from inconsistencies in measuring personal responsibility. Therefore, measuring personal responsibility must be adapted to its cultural context. Spurred by these reasons, we developed a “Chinese College Student Personal Responsibility Scale” (CCSPRS) based on local theoretical foundations. Furthermore, we conducted a preliminary exploration using the new scale, examining the correlations between college students’ responsibility, trust propensity, and prosocial behavior tendencies.

**Methods:**

The initial version of the scale was subjected to item analysis, exploratory factor analysis (EFA), and confirmatory factor analysis (CFA) to form the formal version of the scale. A total of 1,038 college students were assembled. The reliability and validity of the scale were also analyzed. We collected data using the proposed CCSPRS, Interpersonal Trust Scale, and Prosocial Tendencies Measure Questionnaire and obtained 301 valid questionnaires.

**Results:**

The scale’s reliability and validity indicators met the development requirements, and the investigation revealed that women students scored significantly higher in responsibility than men students. Additionally, the responsibility scores were relatively high in the first and fourth years and low in the second and third years, presenting an approximate U-shaped trend. Besides, the college students’ personal responsibility, trust propensity, and prosocial behavior tendencies were positively correlated.

**Discussion:**

The proposed CCSPRS is an effective tool for measuring personal responsibility among Chinese college students. Additionally, this study analyzed the internal beliefs of individuals and concluded that personal responsibility, prosocial behavior, and trust propensity are closely interconnected, especially the relationship between responsibility and prosocial behavior.

## 1. Introduction

Investigating personal responsibility is a classic and dynamic subject that various researchers have approached differently. Typically, responsibility is associated with contributing to the well-being of others and society and adhering to specific norms. Gough et al. (1952) defined responsibility as the willingness to acknowledge the outcomes of one’s actions, dependability, trustworthiness, and a sense of duty to the team. Berkowitz and Daniels (1964) proposed that individuals who demonstrate social responsibility are inclined to assist others, with or without expecting a reward, once they understand that others depend on them to achieve their objectives. Schlenker et al. (1994) contended that responsibility is a psychological adhesive that ties together prescriptions, occurrences, and identity. On the other hand, Starrett (1996) viewed social responsibility as an attitude and behavioral pattern, highlighting that individuals are respectable members of their society or community. In conclusion, the studies mentioned above primarily depicted personal responsibility within the context of social responsibility.

Understanding of responsibility is influenced by the cultural background in which it is embedded. Chinese scholars argued that there are significant differences in the definitions and interpretations of responsibility between the Western and Chinese theoretical contexts (Ye, 2009). For instance, Ren (2008) suggested that responsibility is primarily an internalized value achieved through self-cultivation and self-realization in Confucianism. This understanding of responsibility reflected the typical cultural dependency in China. Some Chinese researchers highlighted that the Chinese people’s interpersonal responsibility focuses on faithfulness and loyalty to others (Zeng and Xia, 2019). Du and Zeng (2018) argued that Chinese individuals assume a proactive moral responsibility, consciously assuming an altruistic responsibility inherent to the “self.” Responsibility in Chinese culture is closely linked to personal and social values, with individual responsibility inherently encompassing responsibility to others and society (Wu, 2013), forming a broad and widely accepted moral responsibility. Guoshu’s theory of the “Four Elements of the Chinese Self” suggested that Chinese individuals have a community-oriented interdependent self with an orientation of responsibility toward others in general (Li and Wang, 2019). Therefore, personal responsibility cannot be separated from social responsibility in Chinese culture, and personal responsibility includes the notion of social responsibility as proposed by previous scholars. The complexity and diversity of cultural influences on the meaning of responsibility are evident in these understandings.

Responsibility is highly valued in traditional Chinese culture, and the construction of personal responsibility affects the formation of the national character of the Chinese people (Ren, 2008). However, responsibility as an inherent personality trait of the Chinese has not received much attention in current personality theory (Zeng and Xia, 2019). On the other hand, the increasing demands for public health (Babcock, 2009) and environmental protection (Bouman et al., 2020) have increased calls for personal responsibility. Therefore, updating the responsibility scale to reflect people’s characteristics in terms of responsibility is important. Indeed, developing and validating a new scale for personal responsibility provides a more effective understanding of individual responsibility in contemporary society. Furthermore, developing the new scale based on locally grounded theory can better reflect the psychological structure of responsibility under a certain cultural background and serve as a measurement tool for further personal responsibility research. Since personal responsibility is related to prosocial tendencies, such as trust and altruism, accurately measuring personal responsibility will assist in understanding the relationship between the variables related to personal prosocial behaviors.

The complexity and ambiguity of the concept of personal responsibility have led to a lack of authoritative tools for measuring it. Indeed, different understandings of personal responsibility resulted in different tools measuring different aspects of responsibility. Only a few scales have been specifically developed for measuring personal responsibility, which lack updated, widely used adult scales. According to Berkowitz and Daniels (1964), scales for measuring social responsibility may not apply to all cultures, with their scale relying on traditional American core cultural values that align with Protestant ethics. Currently, the most widely used measurement tools are measures of conscientiousness in the Big Five personality traits (Jackson et al., 2009, 2010; Roberts et al., 2014).

However, the results of the Big Five personality traits are also influenced by cultural background. Studies revealed that even when the same study used the Big Five personality traits to measure conscientiousness in different Western countries, the results varied significantly (Klimstra et al., 2011; Anusic et al., 2012). Cultural differences between Chinese and Western societies may lead to variations in personality traits (Zeng and Xia, 2019), and therefore, it is challenging to apply questionnaires developed for other cultures, such as the Big Five personality traits questionnaire, to Chinese populations. Consequently, it is inappropriate to use “conscientiousness” as a direct substitute for “responsibility” when applying the Big Five in studies in China. Thus, localized measurement tools are necessary to investigate the concept of responsibility in Chinese culture.

From an individual psychological perspective, responsibility is mainly understood as a personality trait (Jiang and Pang, 2000; Peng, 2003; Tan and Qin, 2005; Zhang and Sun, 2006; Wu and Huang, 2012). For instance, Wu and Huang (2012) claimed that responsibility is an important personality trait, an individual’s conscious awareness and active behavior tendency to fulfill responsibilities based on their cognitive level. He and Huang (2017b) argued that interpersonal responsibility may be a cross-situational personality trait in Confucian culture. As a result, internalized responsibility may grow into a solid psychological structure represented in personality traits that place a high value on commitment, others, and social values. It may also lead to establishing ideas that support social development in cognition and prosocial behavior tendencies.

Despite a wealth of research in the field, there is no agreement on the structure and dimensions of responsibility. Existing studies proposed various dimensions, such as responsibility cognition, emotion, will, and behavior (Zhang, 1998; Tan and Qin, 2005; Li and Ye, 2010). Building upon this foundation, Wu and Huang (2012) utilized grounded theory to develop a localized psychological responsibility structure based on in-depth individual interviews. They identified three dimensions: responsibility cognition, sense of responsibility, and responsibility behavior.

In recent years, the development of Chinese college students’ responsibility questionnaires has mainly relied on bottom-up explorations to identify the three dimensions (Hu and Dai, 2015; Liu et al., 2020; Tao and Cheng, 2020), with some focusing on interpersonal responsibility (He and Huang, 2017a; Zeng and Xia, 2019), or responsibilities toward specific objects such as the environment or the country (Hu and Dai, 2015; Liu et al., 2020). However, there was a lack of top-down construction of a personal responsibility scale based on local trait theory. This study used the three-factor model of responsibility psychological structure of Chinese individuals derived from Wu and Huang (2012) and proposed Research Question 1: Constructing a “Chinese College Student Personal Responsibility Scale” based on a localized theoretical framework.

Socialization of responsibility is essential in developing prosocial behaviors across various cultures (Ochs and Izquierdo, 2009). Prosocial behavior refers to voluntary actions undertaken to benefit others, groups, or society, requiring time, energy, and resources (Schroeder and Graziano, 2015). Several studies have suggested that developing social responsibility is associated with prosocial motivation (Gough et al., 1952; Harris, 1957; Such and Walker, 2004). Previous research has shown that responsibility has a positive correlation with prosocial behavior (Gough et al., 1952; Harris, 1957; Berkowitz and Daniels, 1964; Rushton, 1982; Gore et al., 2009; Xu and Ma, 2013; Li et al., 2022) and a significant positive relationship with altruistic behavior within prosocial behavior (Zhong and Guo, 2008; Li and Ye, 2009; Yu et al., 2014; Li et al., 2022). Individual norm theory suggests that social responsibility norms promote helping or altruistic behavior among individuals (Berkowitz and Daniels, 1964; Schwartz, 1975).

Responsibility positively correlates with trust propensity, representing an individual’s predisposition to trust others, reflecting a generalized expectation of their trustworthiness (Rotter, 1967; Mayer et al., 1995). Prior research within the Chinese cultural context demonstrated a significant correlation between an individual’s trust and sense of responsibility (Hu et al., 2014). Interpersonal responsibility, in particular, exerted a longitudinal positive influence on an individual’s interpersonal trust, independent of the Big Five personality traits, suggesting that interpersonal responsibility may possess distinct socio-psychological functions (Zeng and Xia, 2019). Previous studies examining the relationship between the Big Five personality traits and trust propensity among Chinese university students found no significant correlation between conscientiousness and trust propensity (Xu et al., 2011). Therefore, it becomes imperative to employ localized measurement tools in China to explore the impact of personal responsibility on trust propensity. While previous research primarily focused on relationship-oriented dimensions of responsibility (VanDellen and Baker, 2011; He and Huang, 2017a; Zeng and Xia, 2019; Li et al., 2022), this study investigated whether responsibility, as a personality trait at the individual belief level, correlates with trust propensity.

Prosocial behavior and altruistic personality encompassed concepts, such as social responsibility and interpersonal trust (Rushton, 1982; Carlo and Randall, 2002), indicate that responsibility, trust, and prosociality may be interconnected within the same belief framework. Furthermore, responsibility and interpersonal trust involve an individual’s relationships with others and groups (Hu et al., 2014), and high levels of responsibility and trust propensity increase engagement in prosocial behaviors. Therefore, this work explored whether the personal responsibility measured by the new responsibility scale is related to trust propensity and prosocial behavior. Based on these considerations, we proposed Research Question 2: Does college students’ responsibility correlate with their trust propensity and prosocial behavior tendencies?

## 2. Development of Chinese college student personal responsibility scale

### 2.1. Method

#### 2.1.1. Participants and procedure

A total of 1,038 valid responses were obtained from randomly sampled college students from multiple universities in different regions. The first batch of scales, consisting of 617 initial surveys, was distributed to Sample 1 and Sample 2. After removing invalid scales with maximum scores on deception items or random answers, a final sample of 585 valid responses was obtained. Among the participants, 238 (40.68%) were men and 347 (59.32%) were women, with an age range of 17 to 27 years and a mean age of 21.70 ± 1.71.

Sample 1 included 292 participants, with 126 (43.15%) men and 166 (56.85%) women, with and age range of 17 to 26 years and a mean age of 21.64 ± 1.76, and was used for item analysis and exploratory factor analysis.

Sample 2 included 293 participants, with 112 (38.23%) men and 181 (61.77%) women, with and age range of 18 to 27 years and a mean age of 21.75 ± 1.66, and was used for confirmatory factor analysis, reliability test, and validity test.

In the second batch, Sample 3 consisted of 532 formal scales, of which 453 were valid. Among the participants in Sample 3, 121 (26.71%) were men, and 332 (73.29%) were women, with an age range of 17 to 23 years and a mean age of 18.64 ± 0.98. Sample 3 was utilized for secondary confirmatory factor analysis and comparison with the Big Five personality traits.

Data collection was conducted using an online survey administered through a web-based platform. Participants were informed of the voluntary nature of their participation and the anonymity of their responses. The study received ethical approval from the institutional ethics committee.

#### 2.1.2. Material

##### 2.1.2.1. Development of the preliminary Chinese college student personal responsibility scale

An initial set of items for the scale was developed based on the three-factor model proposed by Wu and Huang (2012). The item statements were derived from interview texts and modified to ensure concise and unambiguous wording. Expert opinions were sought from psychology professors, doctoral students, and master students with extensive experience in social psychology research. The initial set of items was reviewed and revised based on their feedback, resulting in the preliminary version of the Chinese College Student Responsibility Scale. To assess response quality, deception items were included.

The preliminary version of the scale consisted of a total of 32 items, including 27 responsibility items and five deception items. Among the responsibility items, seven were reverse-scored. To enhance item discrimination and avoid response ambiguity, a 6-point rating scale was used for the scale (1 = strongly disagree, 6 = strongly agree). Participants were instructed to select the rating that best reflects their initial response based on the item content.

##### 2.1.2.2. NEO five-factor inventory

The NEO-FFI (NEO-FFI), consisting of the conscientiousness and agreeableness subscales, was selected for this study, with each subscale comprising 12 items (McCrae and Costa, 2004). The Cronbach alpha coefficients for these two subscales in the current study were 0.83 and 0.60, respectively. It is a five-point Likert scale ranging from 1(disagree strongly) to 5(agree strongly). The questionnaire demonstrated relatively stable reliability and validity and has been widely used in studies on the Big Five personality traits.

#### 2.1.3. Statistical analysis

Descriptive statistics, correlation analysis, inferential tests for group differences, and exploratory factor analysis were performed using SPSS 22.0. For the data from Sample 2, confirmatory factor analysis and assessments of reliability and validity were conducted using AMOS 24.0 and jamovi 2.3.26.

### 2.2. Results

#### 2.2.1. Exploratory factor analysis

All 27 items from the initial questionnaire underwent item analysis and were subsequently subjected to exploratory factor analysis. The exploratory factor analysis of the 27 items was conducted using principal component analysis and varimax rotation. A screening criterion was applied, where items with factor loadings exceeding 0.40 and commonalities above 0.30 were retained. Following this process, 14 items were removed, resulting in a final scale (see Appendix) comprising 13 items [KMO = 0.87, Bartlett’s Test of Sphericity *χ*^2^ = 1560.99 (*df* = 78, *p* < 0.001)]. The Chinese version of the scale was used. The English translation version of the scale is provided in the Appendix.

Three factors were extracted based on parallel analysis (O’connor, 2000) to determine the number of factors. The eigenvalues from the parallel analysis are reported in Table 1, where the first factor comprises six items, reflecting the dimension of responsibility cognition. The second factor comprises three items, reflecting the dimension of a sense of responsibility, and the third factor consists of four items, reflecting the dimension of responsibility behavioral tendency.

**Table 1:** The parallel analysis of initial scale (*N* = 292).

Root	Observed eigenvalues	Random eigenvalues(Means)
1	5.12	1.37
2	1.83	1.27
3	1.27	1.21
4	0.75	1.14
5	0.71	1.09

The first five eigenvalues were reported.

#### 2.2.2. Confirmatory factor analysis

When conducting a confirmatory factor analysis for the structural model of the questionnaire, the ML (Maximum Likelihood) method is commonly employed for continuous data. However, researchers (Li, 2016; Simms et al., 2019; Brauer et al., 2023) revealed that Likert-type scales in psychology are essentially a form of non-continuous data, and it is advisable to use the WLSMV (Weighted Least Squares Mean and Variance adjusted) method for analysis. Thus, this paper used the lavaan package to analyze the data.

In addition to the three-factor model, we considered the possibility of a second-order factor model. Therefore, we compared the three-factor model (model 1) and the second-order factor model (model 2), with Table 2 presenting the confirmatory factor analysis results for both models. The results infer that the first-order three-factor and second-order factors models basically met the requirements for model validation, although the RMSEA of the first-order model was slightly greater than 0.8. Additionally, the first-order three-factor model had better CFI and TLI, but the second-order factor model better suited the practical application of the questionnaire.

**Table 2:** The goodness of fit indexes for the compared model of initial scale (*N* = 293).

	WLSMVχ^2^	*df*	*p*	CFI	TLI	SRMR	RMSEA(90%CI)
Model 1	207	62	<0.001	0.996	0.994	0.043	0.089 (0.076, 0.103)
Model 2	93.1	62	0.006	0.959	0.949	0.038	0.041 (0.022, 0.058)

WLSMVχ^2^ robust weighted least squares Chi-square, df degrees of freedom, CFI, comparative fit index; TLI, Tucker-Lewis index; RMSEA, root mean square error of approximation; CI, confidence interval.

By conducting confirmatory factor analysis on the data from Sample 3, which was obtained from different sources, the model fit (Table 3) results were consistent with those of the initial scale. This indicated that the scale can be consistently validated across different groups of college students.

**Table 3:** The goodness of fit indexes for the second-order factor model of formal scale (*N* = 453).

WLSMVχ^2^	*df*	*p*	CFI	TLI	SRMR	RMSEA(90%CI)
204	62	<0.001	0.977	0.971	0.053	0.071 (0.060, 0.082)

WLSMVχ^2^ robust weighted least squares Chi-square, df degrees of freedom, CFI, comparative fit index; TLI, Tucker-Lewis index; RMSEA, root mean square error of approximation; CI, confidence interval.

#### 2.2.3. Reliability and validity test

The item parameters for all four samples are shown in Table 4.

**Table 4:** Item parameters for each sample.

	Sample 1 (*N* = 292)			Sample 2 (*N* = 293)			Sample 3 (*N* = 453)			Sample 4 (*N* = 301)		
Items	Mean (SD.)	CITC	Loadings	Mean (SD.)	CITC	Loadings	Mean (SD.)	CITC	Loadings	Mean (SD.)	CITC	Loadings
C1	4.77 (1.11)	0.58	0.80	4.85 (1.06)	0.60	0.77	5.34 (0.95)	0.34	0.54	5.18 (0.82)	0.44	0.65
C2	4.87 (1.09)	0.61	0.85	4.92 (1.00)	0.62	0.77	5.28 (0.95)	0.50	0.71	5.19 (0.90)	0.52	0.76
C3	4.60 (1.12)	0.56	0.72	4.69 (1.11)	0.59	0.74	4.80 (1.12)	0.43	0.63	4.91 (0.99)	0.41	0.63
C4	4.40 (1.22)	0.46	0.64	4.61 (1.13)	0.51	0.63	4.89 (1.19)	0.34	0.54	4.69 (1.14)	0.29	0.47
C5	4.66 (1.02)	0.58	0.76	4.76 (1.02)	0.60	0.74	5.21 (0.95)	0.29	0.46	4.98 (0.85)	0.44	0.62
C6	4.78 (1.24)	0.58	0.73	4.91 (1.08)	0.62	0.78	5.36 (1.05)	0.42	0.69	5.24 (0.95)	0.47	0.65
S1	4.41 (1.30)	0.59	0.80	4.43 (1.23)	0.57	0.79	4.72 (1.02)	0.36	0.46	4.92 (0.85)	0.30	0.46
S2	4.47 (1.24)	0.59	0.83	4.45 (1.23)	0.58	0.83	4.73 (1.09)	0.43	0.61	4.89 (0.88)	0.52	0.73
S3	4.40 (1.20)	0.60	0.83	4.42 (1.33)	0.59	0.83	5.06 (1.22)	0.22	0.37	4.82 (0.99)	0.50	0.71
B1	3.89 (1.41)	0.41	0.68	3.86 (1.44)	0.39	0.59	4.90 (1.31)	0.37	0.69	4.07 (1.47)	0.39	0.59
B2	4.23 (1.55)	0.54	0.89	4.32 (1.53)	0.52	0.81	5.36 (1.06)	0.43	0.76	4.81 (1.34)	0.50	0.77
B3	3.88 (1.26)	0.36	0.58	3.79 (1.25)	0.40	0.61	4.36 (1.18)	0.34	0.56	3.94 (1.22)	0.39	0.53
B4	3.93 (1.25)	0.48	0.69	4.01 (1.25)	0.53	0.77	4.29 (1.08)	0.35	0.56	4.19 (1.12)	0.40	0.59

C stands for responsibility cognition, S stands for sense of responsibility, B stands for responsibility behavior, CITC corrected item-total correlations.

Internal consistency reliability analysis revealed that both personal responsibility (Cronbach’s α = 0.87; McDonald’s ω = 0.88) and its three sub-dimensions, namely responsibility cognition (Cronbach’s α = 0.88; McDonald’s ω = 0.88), sense of responsibility (Cronbach’s α = 0.85; McDonald’s ω = 0.86), and responsibility behavioral tendency (Cronbach’s α = 0.79; McDonald’s ω = 0.79), exhibited good internal consistency.

Composite reliability (CR) and average variance extracted (AVE) values (Table 5) were calculated to assess the internal structural validity of the scale. The CR values exceeding 0.8 and AVE values exceeding 0.5 indicated good internal reliability and convergent validity of the model.

**Table 5:** CR and AVE for formal scale (*N* = 293).

	CR	AVE
Personal responsibility	0.95	0.59
Responsibility cognition	0.88	0.55
Sense of responsibility	0.87	0.69
Responsibility behavioral tendency	0.84	0.57

CR, composite reliability; AVE, average variance extracted.

The correlation analysis revealed that the correlations between each dimension and the total score ranged from 0.72 to 0.83 (Table 6), indicating a high degree of correlation. The correlations between the dimensions ranged from 0.33 to 0.51, indicating a moderate degree of correlation. These results suggested that the dimensions of the scale were both interrelated and independent of each other.

The results of the Fornell-Larcker criterion (FLC) test (Table 6; Fornell and Larcker, 1981) indicated that the value for the dimension of responsibility cognition was greater than the correlation coefficients between responsibility cognition and other dimensions. Similarly, the value for the sense of responsibility was greater than the correlation coefficients between sense of responsibility and other dimensions. Additionally, the value for the dimension of responsibility behavioral tendency was greater than the correlation coefficients between responsibility behavioral tendency and other dimensions. These findings suggested that the three dimensions exhibited good discriminant validity, as they demonstrated stronger associations within their respective dimensions compared to the associations between different dimensions.

**Table 6:** Correlation analysis of formal scale scores and Fornell-Larcker criterion analysis (*N* = 293).

	Responsibility cognition	Sense of responsibility	Responsibility behavioral tendency
Responsibility cognition	0.74(FLC)		
Sense of responsibility [95%CI]	0.51** [0.43 ~ 0.59]	0.83(FLC)	
Responsibility behavioral tendency [95%CI]	0.33** [0.20 ~ 0.45]	0.34** [0.23 ~ 0.45]	0.76(FLC)
Personal responsibility [95%CI]	0.83** [0.78 ~ 0.87]	0.75** [0.71 ~ 0.79]	0.72** [0.65 ~ 0.78]

FLC, Fornell-Larcker criterion test; the values in the table represent the square root of the average variance extracted (AVE) for each construct; *p < 0.05, **p < 0.01; CI confidence interval.

The Heterotrait-Monotrait ratio (HTMT) analysis revealed that the HTMT value between responsibility cognition and sense of responsibility was 0.59, between responsibility cognition and responsibility behavioral tendency was 0.39, and between sense of responsibility and responsibility behavioral tendency was 0.42. These values were all below the threshold of 0.85, indicating that there existed discriminant validity between these dimensions.

#### 2.2.4. Contrast with the Big Five personality

The results of the correlation analysis between personal responsibility and its dimensions, as well as agreeableness and conscientiousness in Sample 3, were presented in Table 7. The findings indicated a moderate correlation between responsibility, its dimensions, agreeableness, and conscientiousness among Chinese college students.

**Table 8:** Correlation analysis between responsibility, trust, and prosocial behavior (*N* = 301).

	1	2	3	4	5
1 Personal responsibility					
2 Trust propensity [95%CI]	0.24^**^ [0.13 ~ 0.35]				
3 Prosocial behavior tendencies [95%CI]	0.37^**^ [0.26 ~ 0.46]	0.23** [0.12 ~ 0.33]			
4 Responsibility cognition [95%CI]	0.82^**^ [0.79 ~ 0.86]	0.10 [0.01 ~ 0.20]	0.27** [0.17 ~ 0.37]		
5 Sense of responsibility [95%CI]	0.71^**^ [0.64 ~ 0.76]	0.18** [0.07 ~ 0.29]	0.21** [0.10 ~ 0.32]	0.48** [0.39 ~ 0.57]	
6 Responsibility behavioral tendency [95%CI]	0.80^**^ [0.76 ~ 0.84]	0.28** [0.18 ~ 0.38]	0.35** [0.23 ~ 0.46]	0.39** [0.30 ~ 0.49]	0.38** [0.28 ~ 0.48]

*p < 0.05, **p < 0.01; CI, confidence interval.

A t-test was conducted to compare the levels of personal responsibility between men and women in Sample 3. The results revealed a significant difference (*t* = −2.48, *p* = 0.014, Cohen’s *d* = 0.26, 95%CI[−2.92 ~ −0.34]) in overall personal responsibility scores between men (53.61 ± 6.62) and women (55.24 ± 6.04). Specifically, there was a significant difference in the dimension of responsibility behavior inclination (men 16.83 ± 4.01; women 17.82 ± 3.78; *t* = −2.43; *p* = 0.016; Cohen’s *d* = 0.25; 95%CI [−2.58 ~ −0.87]). However, when examining conscientiousness, no significant gender difference was found, although the mean score for men (44.74 ± 6.60) was higher than that for women (43.93 ± 6.39).

## 3. The relationship between personal responsibility, trust propensity, and prosocial behavior tendencies of college students

### 3.1. Method

#### 3.1.1. Participants and procedures

A random sample of 355 was distributed among college students from various regions. After removing invalid questionnaires that contained maximum scores on deception items or random responses, a total of 301 (sample 4) valid responses were obtained. Among them, there were 116 men (38.50%) and 185 women (61.50%), with an age range of 18 to 27 and a mean age of 21.382 ± 1.484. The scale was distributed through an online platform, with participants being informed about the voluntary nature of their participation. The responses were collected anonymously. The collected data from the questionnaire were analyzed and processed using SPSS 22.0.

#### 3.1.2. Measures

##### 3.1.2.1. Chinese college student personal responsibility scale

The CCSPRS (CCSPRS) developed above. The scale contains 13 items divided into three dimensions: responsibility cognition, sense of responsibility, and responsibility behavioral tendency. In this study, Cronbach’s α was 0.79 for the scale.

##### 3.1.2.2. Interpersonal trust scale

The Chinese version of the Interpersonal Trust Scale (ITS) compiled by Rotter (1967) was revised by Wang et al. (1993). It was used to measure trust propensity, which is commonly used in the field (Colquitt et al., 2007). Colquitt et al. (2007) noted that although Rotter’s scale is named the “Interpersonal Trust Scale,” its conceptual meaning and measurement content actually reflect trust propensity. The scale consists of 25 items rated on a 5-point Likert scale. Higher total scores indicate a higher level of trust propensity in individuals. Cronbach’s α was 0.60 for this scale in this study.

##### 3.1.2.3. Prosocial tendencies measure questionnaire

The Chinese version of the Prosocial Tendencies Measure Questionnaire (PTMQ) was revised by Cong (2014) based on the questionnaire compiled by Carlo and Randall (2002). The questionnaire consists of 23 items rated on a 5-point Likert scale, with 12 items being reverse scored. The questionnaire assesses six dimensions: openness (four items), anonymity (five items), altruism (five items), compliance (two items), emotionality (four items), and urgency (three items). Higher total scores indicate a higher level of prosocial orientation. In this study, Cronbach’s α was 0.89 for this questionnaire.

### 3.2. Results

The results of the survey on the current status of college students’ personal responsibility showed that the average score for overall personal responsibility was 4.76 ± 0.56, the average score for responsibility cognition was 5.03 ± 0.60, the average score for sense of responsibility was 4.88 ± 0.69, and the average score for responsibility behavioral tendency was 4.76 ± 0.56.

A t-test was conducted to examine the differences in the scores of personal responsibility between man and woman students (*t* = −2.79; *p* = 0.006; Cohen’s *d* = 0.33; 95%CI [−4.09 ~ −0.71]). The results revealed a significant difference, with man students (60.37 ± 7.56) scoring lower than woman students (62.77 ± 7.05). Significant differences were found in the dimension of responsibility behavior tendency, indicating that man (15.96 ± 3.90) and woman (17.68 ± 3.28) students differed significantly in their scores in this particular dimension (*t* = −3.96; *p <* 0.001; Cohen’s *d* = 0.48; 95%CI[−2.58 ~ −0.87]).

A one-way analysis of variance (ANOVA) was conducted to examine the differences in personal responsibility scores among students from different academic years [*F*_(3, 297)_ = 3.15; *p* = 0.025; η^2^ = 0.03]. The results revealed a significant difference in the overall personal responsibility scores across academic years. *Post-hoc* comparisons showed that freshmen (63.15 ± 7.75) had significantly higher responsibility scores than juniors (60.22 ± 7.35; *p* = 0.019; Cohen’s *d* = 0.39; 95%CI [0.48 ~ 5.38]). And seniors (62.71 ± 6.86) had higher scores than juniors (*p* = 0.018; Cohen’s *d* = 0.35; 95%CI [0.43 ~ 4.55]) as well. Furthermore, significant differences were observed among academic years in the dimension of responsibility behavior tendency. Specifically, seniors (17.70 ± 3.63) scored significantly higher than sophomores (15.97 ± 3.91; *p* = 0.009; Cohen’s *d* = 0.48; 95%CI [0.43 ~ 3.03]) and juniors (16.26 ± 3.60; *p* = 0.005; Cohen’s *d* = 0.35; 95%CI [0.43 ~ 2.46]) in this dimension.

According to the correlation analysis presented in Table 8, it was found that there were significant positive correlations among personal responsibility, interpersonal trust, and prosocial behavior tendencies for each pair.

**Table 7:** Correlation analysis between formal scale scores and Big Five personality scores (*N* = 453).

	Responsibility cognition	Sense of responsibility	Responsibility behavioral tendency	Personal responsibility
Agreeableness [95%CI]	0.24** [0.14 ~ 0.33]	0.33** [0.24 ~ 0.41]	0.43** [0.35 ~ 0.50]	0.41** [0.33 ~ 0.49]
Conscientiousness [95%CI]	0.32** [0.24 ~ 0.41]	0.34** [0.25 ~ 0.42]	0.43** [0.35 ~ 0.50]	0.47** [0.39 ~ 0.54]

*p < 0.05, **p < 0.01; CI, confidence interval.

## 4. Discussion

This study discussed the importance and uniqueness of responsibility in Chinese culture and analyzed the necessity of developing a personal responsibility measurement tool based on local theories. The current study constructed a “Chinese College Student Personal Responsibility Scale” based on the local grounded theory of the psychological structure of responsibility. Through item analysis, exploratory factor analysis, confirmatory factor analysis, and tests of reliability and validity, the developed scale demonstrated good reliability and validity, allowing it to measure personal responsibility among Chinese college students. Furthermore, a preliminary investigation and exploration of personal responsibility were conducted, examining the correlations between personal responsibility, trust propensity, and prosocial behavior tendencies among college students. The study confirmed that personal responsibility positively correlates with prosocial behavior tendencies and trust propensity.

### 4.1. Measurement tools adapted for Chinese culture to evaluate personal responsibility

As a personality trait in Chinese culture, personal responsibility requires measurement tools adapted to the cultural context. Previous research has indicated that interpersonal responsibility plays a positive role that cannot be explained solely by the Big Five personality traits (Shen et al., 2016), as its influence is independent of the Big Five. Besides, a cross-cultural study conducted in the United States and Japan (Dunkel, 2013) found that conscientiousness was positively correlated with independent self-construal. This suggests conscientiousness is likely to differ from personal responsibility, which emphasizes collective self-values. Therefore, it is necessary to explore the concept of responsibility in the Chinese cultural context using a locally grounded theoretical framework. Hence, this study developed a “Chinese College Student Personal Responsibility Scale” based on this framework. The scale demonstrated satisfactory measurement properties, and the results demonstrated a correlation between personal responsibility and conscientiousness in the range of 0.3 to 0.4, indicating a relationship with conscientiousness while also exhibiting some level of independence, reflecting the possibility that personal responsibility in Chinese culture may not align entirely with conscientiousness.

### 4.2. Current situation of personal responsibility of college students in China

A gender-based analysis of the results revealed that women students had significantly higher scores in overall personal responsibility and the responsibility behavior dimension than men students. These findings were consistent with previous research on social responsibility in China (Liu et al., 2011). Regarding the gender differences in the responsibility behavior dimension, other researchers have also found that women college students scored significantly higher than men students in the action stage of social responsibility (Wei, 2014). Cross-cultural studies have generally shown that women exhibit higher levels of responsibility than men (Schmitt et al., 2008). Research conducted in Western organizations has also found that women exhibit significantly higher social responsibility than men (Alonso-Almeida et al., 2017), and a study conducted in Thailand similarly found that women college students had higher levels of social responsibility than men students (Sosik et al., 2017).

At the same time, we also found some inconsistent results. Unlike studies that used the Big Five conscientiousness measure and observed that men scored higher in conscientiousness than women (Li and Chen, 2015; Shi, 2018), our study, although presented a similar trend where men’s conscientiousness scores were higher than women’s (although not statistically significant), women’s responsibility scores were significantly higher than men’s. This result suggests that we should exercise caution in equating conscientiousness with responsibility within Chinese culture, and further research is required to delve deeper into the relationship between responsibility and conscientiousness. Therefore, it is important not to confuse or directly apply different measurement tools interchangeably.

An analysis of the grade-level results revealed that first-year students had higher personal responsibility scores than sophomores and juniors, which confirms current research findings (Tao and Zhu, 2014). The distribution of personal responsibility scores across different grade levels shows higher scores in first-year students and seniors compared to sophomores and juniors, which aligns with previous studies on social responsibility in college students. These studies revealed a pattern of high scores in first-year students and seniors and lower scores in sophomores and juniors, resembling a U-shape (Luo, 2007; Zhao et al., 2010). Besides, some studies have indicated that first-and second-year students exhibit significantly higher comprehension of social responsibility than juniors and seniors (Wei, 2014).

However, some studies found no significant differences in social responsibility among different grade levels of university students (Liu et al., 2011). These variations in findings may be attributed to the psychological state of university students. Indeed, first-year students enter a new stage of their lives with enthusiasm and expectations, hoping to be more involved in university affairs and take on more responsibilities. Tao and Zhu (2014) suggested that sophomores, juniors, and seniors may experience more psychological conflicts than first-year students. This is because senior students are about to enter society and consider that their personal career development may require assuming new roles and engaging in new experiences, thereby increasing their responsibility. However, there is a chance that the rise in psychological conflicts will also have an effect. Therefore, the reasons for the grade differences need to be further explored.

### 4.3. The correlations between personal responsibility, trust propensity, and prosocial behavior tendencies

We find that personal responsibility positively correlates with trust propensity, which confirms current research findings (Shen et al., 2016; Zeng and Xia, 2019). Prior studies indicated that individuals with high interpersonal responsibility may be more likely to perceive others as trustworthy (Zeng and Xia, 2019). Individuals with high levels of responsibility have a stronger sense of obligation and responsibility in their cognition, a more positive attitude toward fulfilling their responsibilities in their emotions, and a greater tendency to engage in responsible behavior. According to the social projection theory, individuals tend to project their beliefs and behaviors of adhering to social norms onto others, leading them to perceive others as more trustworthy (Krueger et al., 2008).

Higher personal responsibility correlates with higher prosocial behavior, which confirms the current literature (Carlo and Randall, 2002; Xu and Ma, 2013). Research has shown that organizations exhibit more prosocial behavior when those in power perceive their authority as a responsibility (Tost, 2015; De Wit et al., 2017). According to the sociocultural motives perspective (Gebauer et al., 2014), responsibility triggers assimilation to society’s social and cultural norms. In Chinese culture, which emphasizes collectivism and the social value of the individual, personal responsibility promotes prosocial behavior advocated by social norms. The individual norm theory (Schwartz, 1975) or social norm theory (Berkowitz and Daniels, 1964) explains that internalizing or activating social rules leads to prosocial behavior and personal responsibility resulting from internalized social norms. When individuals perceive themselves as responsible for others and society, they are more likely to attend to the needs of others, adhere to social norms, and engage in altruistic prosocial behavior.

In this study, personal responsibility as a personality trait had a stronger correlation with prosocial behavior tendencies than trust propensity. Previous research has primarily focused on the influence of interpersonal trust on prosocial behavior, emphasizing the interaction between individuals and others or the environment. In contrast, this study emphasized the internal belief relationships within individuals. The relationship among personal responsibility, trust propensity, and prosocial behavior tendencies is worth further exploring.

As a result, prior research did not adequately account for the importance of personal responsibility. Cross-cultural studies between the United States and Japan conducted by Dunkel (2013) revealed a positive correlation between conscientiousness and independent self-construal, indicating that the concept measured by conscientiousness does not necessarily reflect the internalization of social norms. Therefore, it is challenging to discover the impact of personal responsibility on prosocial behavior in a collectivistic culture. Thus, in this study, personal responsibility, as a reflection of internalized social norms and values, may have greater correlations with prosocial behavior than an individual’s trust propensity.

### 4.4. Limitations and prospects

This study focused on individuals adopting Chinese culture, and it would be beneficial to conduct cross-cultural research to explore the individual differences in personal responsibility and their impact on prosocial behavior in different cultures. Future research could investigate the differences in personal and social responsibility between these cultures in greater detail. Additionally, the scale was only administered to college students, and it would be valuable to expand the sample to different populations to examine the applicability of the scale across diverse groups or modify it specifically for different populations to enhance its generalizability.

Furthermore, this study only examined the correlational relationships between personal responsibility, trust propensity, and prosocial behavior. Further investigation into the interplay between these three factors would be valuable in future research. On the other hand, it would be interesting to include other relevant variables, such as fairness and honesty, and explore their relationships. Building upon this study’s findings, the experimental research design may provide further insights into the probable causal relationship between personal responsibility and prosocial behavior.

## 5. Conclusion

This study developed the ‘Chinese College Student Personal Responsibility Scale’ based on Chinese indigenous theory and demonstrated its good reliability and validity, making it a useful tool for measuring the personal responsibility of Chinese college students. The preliminary exploration using the scale revealed a significant positive correlation between personal responsibility and trust propensity and between personal responsibility and prosocial behavior tendencies.

## Data availability statement

The original contributions presented in the study are included in the article, further inquiries can be directed to the corresponding author.

## Ethics statement

The studies involving humans were approved by School of Education, Soochow University. The studies were conducted in accordance with the local legislation and institutional requirements. The participants provided their written informed consent to participate in this study.

## Author contributions

YR: conceptualization, methodology, investigation, formal analysis, data curation, and writing – original draft. JW: conceptualization, writing – review & editing, supervision, project administration, and funding acquisition. HQ: formal analysis, investigation, data curation, writing – original draft. All authors contributed to the article and approved the submitted version.

## Appendix

Chinese College Student Personal Responsibility Scale (CCSPRS).

1. I am capable of putting myself in other people’s shoes.

2. It is wise not to make promises I cannot keep.

3. I might make excuses when I am accused of my mistake.

4. If I know the request is inappropriate, it is reasonable to refuse to help the other person.

5. I try my best to keep my promise even when it is challenging.

6. I think that more promises should come with more work accordingly.

7. I would cross the street directly without traffic, even if the pedestrian lights are red.

8. I think that it is not appropriate to make promises thoughtlessly.

9. I would worry about how my actions might affect the group l belong to.

10. I may do the same if the people in front of me are not queuing in order.

11. I think that it is wrong to shirk responsibility.

12. I will not make thoughtless promises if the appeal puts me in a tough spot.

13. When strangers ask me for help, I tend to do it (without care) as fast as possible.

Responsibility cognition = 2, 4, 6, 8, 11, 12.

Sense of responsibility = 1, 5, 9.

Responsibility behavioral tendency = 3, 7, 10, 13.

Note: 3, 7, 10, 13 are reverse scoring items.
